# Utilization of Native CRISPR-Cas9 System for Expression of Glucagon-like Peptide-1 in *Lacticaseibacillus paracasei*

**DOI:** 10.3390/foods14101785

**Published:** 2025-05-17

**Authors:** Mumin Zheng, Shuwen Zhang, Yunna Wang, Ning Xie, Xiaodan Wang, Jiaping Lv, Xiaoyang Pang, Xu Li

**Affiliations:** Key Laboratory of Agro-Products Quality and Safety Control in Storage and Transport Process, Ministry of Agriculture and Rural Affairs, Institute of Food Science and Technology, Chinese Academy of Agricultural Sciences, Beijing 100193, China; zmm1998vip@163.com (M.Z.); zswcaas@hotmail.com (S.Z.); wang_yn92@163.com (Y.W.); xiening@caas.cn (N.X.); prettyshoot@126.com (X.W.); lvjiapingcaas@126.com (J.L.)

**Keywords:** *Lacticaseibacillus paracasei*, native CRISPR-Cas9, glucagon-like peptide-1 (GLP-1), genome editing, heterologous expression

## Abstract

Type 2 diabetes is one of the main causes of cardiovascular diseases, kidney diseases, and visual impairments, posing a global healthcare challenge. The current treatment of this disease, involving glucagon-like peptide-1 (GLP-1), is faced with problems such as frequent injections and plasmid instability. In this study, we used the native clustered regularly interspaced short palindromic repeats-CRISPR-associated protein 9 (CRISPR-Cas9) system of *Lacticaseibacillus paracasei* to develop a novel, genetically stable, and orally administrable strain expressing human GLP-1. Integration and subsequent expression of glp-1 gene were confirmed by genomic sequencing, qPCR, and Nano LC-MS. The engineered strain demonstrated stable genomic integration and sustained high-level expression of GLP-1 over multiple generations. This innovative approach provides a promising strategy for the oral delivery of therapeutic peptides, potentially enhancing patient compliance and improving the treatment of diabetes and other chronic diseases requiring peptide-based therapies.

## 1. Introduction

Diabetes is a global health crisis, with its prevalence having nearly doubled over the past two decades, causing serious trouble in public health systems worldwide [1]. Type 2 diabetes is a major concern due to its common complications, including cardiopathy and nephropathy, etc. [2]. These far-reaching consequences have promoted abundant research into alternative therapeutic strategies that can offer patients an enhanced quality of life [3]. Existing therapeutic options include exercise schedule, dietary modification, insulin injection, and oral hypoglycemic agents. However, many of these approaches not only increase the patients’ physical and financial burdens but are also ineffective in maintaining long-term glycemic control [3]. Therefore, it is an urgent need to develop novel treatment modalities addressing medical needs and improving patient well-being.

Drug therapy is the most conventional method for controlling blood glucose levels. Distinct pharmacological mechanisms, such as insuCRISPlin replacement, hypoglycemic agents, glucagon-like peptide-1 (GLP-1) receptor agonists, and Dipeptidyl Peptidase-4 (DPP-4) inhibitors, have been developed, and a variety of commercial medicines have been the widely adopted for type II diabetes patients. Among these, GLP-1 plays a central role in diabetes management. The human proglucagon genes, *glp-1* and *glp-2,* were firstly cloned and sequenced in 1983 [4]. GLP-1 binds to receptors, stimulates insulin secretion and inhibits glucagon secretion in a glucose-concentration-dependent manner [5]. It can also delay gastric emptying and suppress appetite, thereby reducing blood sugar. These mechanisms work together both inside and outside the pancreas to significantly reduce the risk of hypoglycemia due to their glucose-dependent nature [6]. So, GLP-1 is a pivotal regulator for glucose metabolism and could be a potential agent for treating type 2 diabetes. In 2017, Novo Nordisk’s semaglutide (Ozempic^®^ Novo Nordisk, Copenhagen, Denmark), a long-acting GLP-1 receptor agonist, was approved by the U.S. FDA for type II diabetes, marking a breakthrough in GLP-1 therapeutics [7]. Additionally, Eli Lilly’s tirzepatide (Mounjaro^®^ Eli Lilly and Company, Indianapolis, IN, USA), a dual GIP/GLP-1 receptor agonist, received approval in China for type II diabetes in 2024, following its U.S. approval in 2022. Despite these advances, injectable GLP-1 agonists (e.g., semaglutide, tirzepatide) remain the primary clinical option [8]. Although an oral formulation of semaglutide (Rybelsus^®^ Novo Nordisk, Copenhagen, Denmark) was approved in 2019, its limitations in efficacy and bioavailability highlight the ongoing need for more effective oral agents [9].

*Lacticaseibacillus* is a vital probiotic bacterial to human health, with the advantages of regulating intestinal flora, promoting digestion, and enhancing immunity [10]. Notably, several *Lacticaseibacillus* strains have been investigated for glycemic control properties, being promisingly helpful in type II diabetes therapy. Also, *Lacticaseibacillus* shows great potential in the medical field, and is suitable as an oral drug delivery vehicle with multiple advantages, such as being easily cultivated and massively produced, showing good colonization ability in the intestine [11]. Furthermore, *Lacticaseibacillus,* as a safe carrier, could avoid the complicated purification steps, allow the effective constituent to be directly absorbed through mucous membranes, and improve drug palatability and patient compliance [12]. In previous studies, recombinant *Lacticaseibacillus* carried tumor suppressor peptides and targeted cervical cancer cells in HPV-related tumor treatment [13]. Zhang et al. used modified *Lactococcus lactis* as a carrier to actively secrete the antimicrobial peptide Cathelicidin to treat Helicobacter-pylori-infection-related diseases [14]. Similarly, modified *L. lactis* was also used in recent diabetes therapy. Takiishi et al. developed engineered *L. lactis* with heterologous expression of Human Proinsulin and Interleukin-10, which effectively lowered the blood glucose level of NOD mice with newly developed diabetes [15].

In this study, we utilized probiotic *L. paracasei* NMG-13, which is isolated from the traditional fermented food in Inner Mongolia [16]. With its native clustered regularly interspaced short palindromic repeats-CRISPR-associated protein 9 (CRISPR-Cas9) system, *L. paracasei* was tracklessly edited and the humanized *glp-1* gene was successfully integrated into the *L. paracasei* chromosome. Then, qPCR and Nano LC-MS analyses were used to prove the expression of the integral GLP-1 peptide. Additionally, the growth rate, acid and bile salt tolerance, genetic stability and the sustained expression of GLP-1 were tested in edited strain.

## 2. Materials and Methods

### 2.1. Strains, Plasmid, and Culture Conditions

*Escherichia coli* DH5α was used as the cloning host and cultured in Luria Bertani (LB) medium at 37 °C. *L. paracasei* NMG-13 was cultured in the Man Rogosa Sharpe (MRS) medium [17] at 37 °C. The *E. coli*-*L. paracasei* shuttle plasmid pTRST, derived from pTRKH2 (Addgene Inc., Watertown, MA, USA), was constructed by introducing the *pldh*-sgRNA module, LpCas9 scaffold, erythromycin and ampicillin resistance genes, and appropriate restriction sites. The transformants were selected on an agar plate with 100 μg/mL erythromycin for *E. coli* or 10 μg/mL for *L. paracasei*. All plasmids used in this study are shown in Table 1.

### 2.2. Analyses of the CRISPR-Cas System

The distribution of CRISPR-Cas systems in *Lacticaseibacillus* was analyzed using the CRISPRCasFinder and CRISPRdb (https://crisprcas.i2bc.paris-saclay.fr (accessed on 26 September 2021)). The CRISPR array and the Cas proteins of *L. paracasei* NMG-13 were identified using the CRISPRminer web server (http://www.microbiome-bigdata.com/CRISPRminer/ (accessed on 21 October 2021)) [18]. The spacers were extracted from the array and analyzed by CRISPRTarget to predict the Protospacer Adjacent Motif (PAM) sequences [19]. The consensus motif of the PAM sequences was shown by WebLogo (http://weblogo.berkeley.edu/logo.cgi (accessed on 25 October 2021)) [20]. The tracrRNA sequence was predicted based on the interaction between it and crRNA, and the single-guide RNA (sgRNA) was designed according to the crRNA/tracrRNA duplex structure.

### 2.3. Construction of CRISPR-Cas9 Editing Plasmids

Editing plasmid was composed of a sgRNA expression module and two homologous arms. The sgRNA expression module included a promoter P_ldh_ of the lactate dehydrogenase from *L. paracasei* and a designed sgRNA containing a 26-nucleotide (nt) sequence targeting a specific genomic locus of the *L. paracasei*. The two homologous arms (1,000 bp) were from the sequences franking the target locus and were amplified from the genomic DNA.

To construct the editing plasmid, a 30 nt spacer sequence located upstream of the PAM (5′-TGAAA-3′) was selected and designed into the forward primer to amplify the tracrRNA fragment employing the genomic DNA of the *L. paracasei* NMG-13 as the template. The two sgRNAs coding sequences generated were fused with the promoter P_ldh_ sequence by overlap extension PCR to obtain the sgRNA expression cassette. Similarly, the two homologous arms were fused and merged with the sgRNA expression cassette into a 2.4 kb fragment. Then, the fragment was ligated into pTRST through Gibson assembly as vectors of gene-editing plasmid.

In the selection of the insertion fragments, the most abundant fraction of GLP-1 in blood was chosen for the experiments (7–36 aa) [21]. This gene was synthesized by BGI (Beijing, China) and fused with promoter P_ldh_ to generate the donor fragment. The donor fragment and the promoter P_ldh_ were ligated into the vector by Gibson assembly to produce the editing plasmid pTRST-GLP-A and pTRST-GLP-B. The primers required for plasmid construction are shown in Table 2.

### 2.4. Preparation of L. paracasei Competent Cells

A modified protocol was used to prepare competent cells [22]. *L. paracasei* NMG-13 was cultured in MRS medium with 3% glycine until the OD_600_ reached 0.6–0.7. The cultures were transferred into 50 mL sterile centrifuge tubes and incubated in an ice bath for 15 min. The bacterial cells were pelleted by centrifugation at 5000 rpm for 15 min, washed twice with 10% glycerol, and resuspended in the same buffer.

### 2.5. Electrotransformation of L. paracasei

Electroporation was performed using 2 mm cuvettes at 2000 V, 400 Ω, and 25 μF. The gene-editing plasmid pTRST-GLP-A and pTRST-GLP-B were electroporated into *L. paracasei* NMG-13, followed by a 3 h recovery at 37 °C. The transformants were selected on MRS agar supplemented with erythromycin and incubated anaerobically for 72 h.

### 2.6. Colony PCR Verification

PCR screening was conducted using YZ-QR-F targeting the upstream *tpiA* gene and reverse primer YZ-QR-R targeting the inserted *glp-1* gene. Electrophoresis was performed to detect amplified bands. Successful gene editing was confirmed by the presence of a 1616 bp target band, whereas the absence of this band indicated editing failure.

### 2.7. Plasmid Curing

Edited strains were passaged repeatedly in a non-selective MRS medium until *L. paracasei* could not grow in an erythromycin-resistant medium but could grow in a normal MRS medium. The colony genome was extracted. The erythromycin gene was amplified by PCR with wild-type, pre- and post-passaged genomes and gene-editing plasmids for electrophoresis detection.

### 2.8. Stability Testing of Gene-Edited Strains

After activating the gene-edited strain, it was successively passaged 50 times. The passaged strains were sequenced and subjected to RT-qPCR. NanoLC-MS was used to test their genetic stability. In addition, the pH of MRS medium was adjusted to 2.0, 3.0, and 4.0 with hydrochloric acid and autoclaved at 115 °C for 20 min. Taurocholine was added to the MRS medium to achieve the final concentrations of 0, 0.1%, 0.3%, and 0.5%, and then autoclaved at 115 °C for 20 min. Single colonies were cultured and passaged twice (18 h each) for activation. Then, the strains were inoculated into the MRS media adjusted with pH and taurocholine with a final OD_600_ value of 0.2–0.8. The dilution pour plate method was used to count the colonies of lactic acid bacteria (LAB). First, the bacterial liquid was serially diluted; then, an appropriate amount of sample liquid with different dilution degrees was poured onto the plates; and finally, cultivation and colony counting were carried out [23].

### 2.9. RT-qPCR

The total RNA of *L. paracasei* was extracted [24] and reverse-transcribed into cDNA by using a reverse transcription kit (ABclonal RK20433, Wuhan, China). The expression of *glp-1* was quantified using SYBR Green I qPCR, with 16S rRNA as the reference gene. Primers RNA-A-F, RNA-A-R, PD-16S-F, PD-16S-R are shown in Table 2. The data were analyzed and normalized by the 2 ^(∆∆Ct)^ method.

### 2.10. NanoLC-MS

The gene–edited strain for two days was anaerobically cultured and then centrifuged. Subsequently, the supernatant and the pellet were stored separately. The preserved supernatant and the broken bacterial contents were processed with Waters C18 Cartridges for desugarization and desalination. The lyophilized GLP-1 sample was dissolved in a 0.1% formic acid solution, separated by a chromatographic gradient for 60 min, and then detected by a Thermo Orbitrap Fusion Lumos ultra-high resolution mass spectrometer. The high-performance liquid chromatography (HPLC) (Easy nLC 1200, Thermo Fisher Scientific, Waltham, Massachusetts, MA, USA) methodological parameters were as follows: C18 chromatographic column: 3 µm, 100Å, 75 µm × 15 cm; sample volume: 2 μg; mobile phase: A: 0.1% formic acid in water; B: 0.1% formic acid in 80% acetonitrile in water. The raw data were analyzed using the proteomics data analysis software PEAKS Studio X (10.0).

### 2.11. Statistical Analyses

Statistical analyses were conducted using SPSS 22 software. Differences in gene expression levels between the engineered and control strains were evaluated using Student’s *t*-test, with a *p*-value ≤ 0.05 considered statistically significant. All experiments were replicated three times to ensure consistency and reliability of the results.

## 3. Results

### 3.1. Determination of Elements of the Gene-Editing Plasmid

The gene editing of *L. paracasei* was achieved by the native CRISPR-Cas system combined with exogenously introduced guide RNAs. In this study, TGAAA was selected as the PAM site. Benchling (https://www.benchling.com/ (accessed on 10 January 2023)) was used to design sgRNA with the *eno* gene as the target and 5′-TGAAA-3′ as PAM. As shown in Table 3, sgRNA-A and sgRNA-B with higher on-target scores were selected as the experimental sgRNAs.

**Figure 1:**
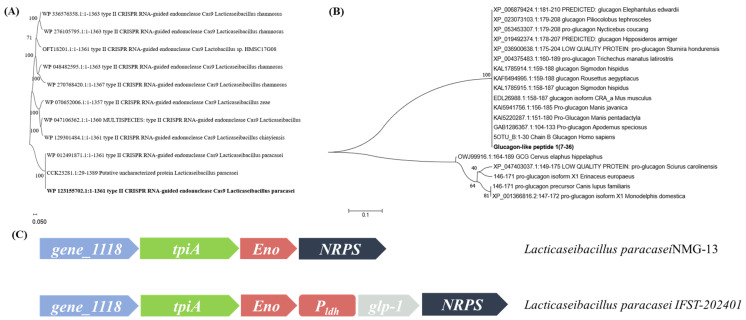
(**A**) The phylogenetic tree drawn using the sequences of Cas9 protein. The phylogenetic tree shows that most of the sequences are more tightly clustered together in the evolutionary tree, These sequences all belong to the type II CRISPR RNA-guided endonuclease Cas9. (**B**) The evolutionary tree was drawn using human GLP-1 protein sequences. The GLP-1 aa sequences of mammals were clustered into a branch with a similarity of 100%, indicating their closest evolutionary relationship. (**C**) A schematic representation of the location of *glp-1* insertion within the genome of *L. paracasei*.

In the early stage, the whole genome of *L. paracasei* (GenBank accession CP002616) was sequenced to characterize its CRISPR-Cas system. The Cas9 orthologous protein was screened and the function of LpCas9 was verified. The regulatory mechanism of the endogenous system was clarified, and the anti-phage function was verified [25]. For identification of homologs of the CRISPR/Cas9 protein in *L. paracasei* NMG-13, BLASTp searches were performed using the native CRISPR/Cas9 in *L. paracasei*. A total of 11 LAB-derived Cas9 homologous proteins were used to construct the phylogenetic tree, as shown in Figure 1A. The results indicated that most of the aa sequences were identified as “type II CRISPR RNA-guided endonuclease Cas9”. This suggests that these sequences are related to the CRISPR-Cas9 system, which has been widely applied in many areas, such as gene editing. Homology analysis confirmed that this protein is also Cas9 in *L. paracasei* NMG-13.

The complete human GLP-1 sequence was retrieved from NCBI. And the most abundant portion (7-36 aa) in human blood was selected, optimized for the codon preference of *L. paracasei* and synthesized by the BGI Genomics company for subsequent experiments. BLASTp searches were performed using human GLP-1 as a query in the NCBI. A total of 20 homologous proteins was used to construct the phylogenetic tree, as shown in Figure 1B. Multiple distinct branches were observed in the phylogenetic tree. The GLP-1 sequences of fish formed a relatively concentrated branch, indicating that its GLP-1 aa sequences were conserved. The GLP-1 aa sequences of mammals were also clustered into a branch with a similarity of 100%, indicating their closest evolutionary relationship. The final location of the knock-in site is shown schematically in Figure 1C.

The *eno* gene encodes glycolytic enzymes. However, this gene is not an essential gene in *L. paracasei* because there are redundant metabolic pathways for glycolysis. Gu et al. confirmed that this strain could still grow normally after knocking out or inserting the foreign gene, indicating that the gene is not an essential gene. In addition, the stability and accuracy of the knock-in site were confirmed by chloramphenicol resistance verification and genotype sequencing [26]. Therefore, the *eno* gene was selected as the knock-in site of the *glp-1* gene in this experiment.

### 3.2. Construction of Gene-Editing Plasmids

To construct the CRISPR-Cas9 system, the Cas9 and sgRNA expression modules were first integrated by a single-plasmid system. The Cas9 gene was optimized with a strong promoter P_ldh_ and paired with an RNA-polymerase-III-dependent promoter for transcription of the sgRNA. The 20 nt targeting sequence of sgRNA and an adapted scaffold can maintain the correct structure of sgRNA for binding to Cas9. Meanwhile, an erythromycin resistance marker was introduced in the plasmid for the screening of positive strains. The sgRNA was engineered based on the 5′-TGAAA-3′ PAM site, and the potential off-target effects were systematically minimized using the CRISPRscan algorithm. Subsequently, electroporation was employed to achieve high-efficiency delivery of CRISPR-Cas9 components into the target bacterial strain.

**Figure 2:**
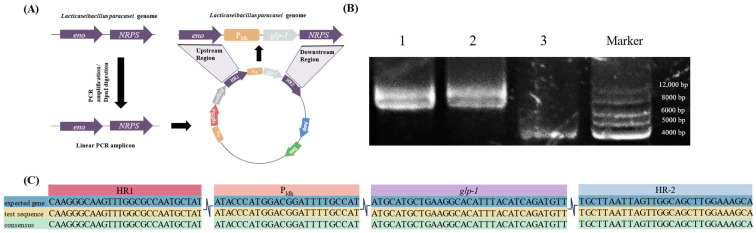
(**A**) The flowchart of plasmid construction. (**B**) Restriction enzyme digestion and electrophoresis verification were performed on the main element regions of the plasmid. Among them, 1 and 2 were gene-edited plasmid digested with NcoI, and 3 was the original modified plasmids digested with NcoI. The target size of bands of 1 and 2 should be 7984 bp, and the target size of band of 3 should be 4891 bp. The electrophoresis bands were in line with the target size. (**C**) Sequence alignment plots of the expected constructed plasmid and the experimental constructed plasmid at the homology arm, promoter and *glp-1* were shown, and the results showed that the plasmid was successfully constructed.

P_ldh_ from *L. paracasei* was also selected to promote the promoter of GLP-1 protein expression. P_ldh_ is a promoter of lactate dehydrogenase. Lactate dehydrogenase is usually highly active and stable in *Lacticaseibacillus* spp. and can efficiently initiate the transcription of downstream genes. The upstream homology arm HR1, promoter P_ldh_, target gene *glp-1*, and downstream homology arm HR2 were assembled into a fragment by reverse PCR, which then was cloned into vector pTRST to construct the gene-editing plasmid. The experiment flowchart is shown in Figure 2A. The constructed plasmids were detected using electrophoresis, and the correct size of target plasmids was observed as shown in Figure 2B. Furthermore, the two constructed plasmids, pTRST-GLP-A and pTRST-GLP-B, were sequenced and compared with the sequence of the original plasmid. The results indicate that the sequences were correct (Figure 2C). These results confirm that the pTRST-GLP-A and pTRST-GLP-B plasmids were successfully constructed.

### 3.3. glp-1 Insertion into L. paracasei NMG-13 

**Figure 3:**
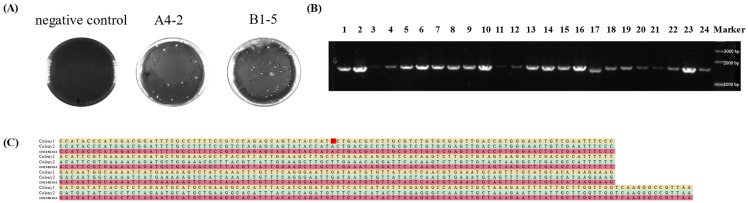
(**A**) The colony growth after plasmid electro transformation of the strains, where A is the negative control, B is the strain edited by the pTRST-GLP-A gene-editing plasmid, and C is the strain edited by the pTRST-GLP-B gene-editing plasmid. (**B**) The results of agarose gel electrophoresis, which showed that the target fragment was successfully integrated into the genome of *L. paracasei*. Combined with the sequencing results, it was found that nineteen of them were positive for colony electrophoresis. Numbers 3, 4, 11, 20, and 21 are false positives. (**C**) The sequence comparison screened out the successful gene-edited strains, and indicated that there were false positives in the picked colonies. A single base mutation was found, which was highlighted in red in the figure, whereas the sequences of the successful gene-edited strains were consistent with the expected results.

The two gene-editing plasmids, pTRST-GLP-A and pTRST-GLP-B, were electrotransformed into *L. paracasei* NMG-13, yielding 40 and 33 erythromycin-resistant colonies, respectively (Figure 3A). By PCR verification, 15 positive colonies for pTRST-GLP-A were obtained with the positive rate of 37.5%, while only four positive colonies for pTRST-GLP-B were obtained with the positive rate of 12.12% (Figure 3B). To further verify the accurate insertion, the fragments of upstream, downstream, promoter P_ldh_ and *glp-1* genes in the 19 positive colonies were sequenced. Of 15 positive colonies of pTRST-GLP-A, the sequences of 14 colonies were completely consistent with expected sequences. One site mutation was observed in another positive colony (Figure 3C). All four positive colonies of pTRST-GLP-B were correct in the sequences compared to the target gene. These results suggest that the *glp-1* gene could be successfully and efficiently integrated into the genome of *L. paracasei* NMG-13. Colony A4-2 with a high expression of *glp-1* was selected for the follow-up experiments.

### 3.4. The Expression of GLP-1 in L. paracasei

**Figure 4:**
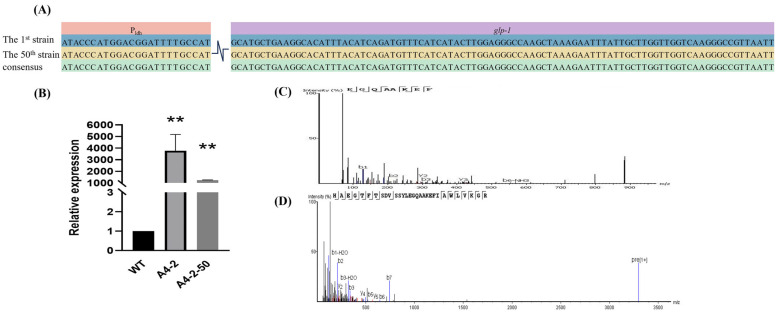
(**A**) A comparison of the sequencing results before and after 50 generations of transmission. (**B**) The extraction of *L. paracasei* RNA for analysis of mRNA expression by RT-qPCR to detect the 1st generation and the 50th generation of transmission. (**C**) The characteristic fragment EGQAAKEF was detected in the first generation of the engineered strain by Nano LC-MS analysis, but not in the WT strain. (**D**) Secondary mass spectrometry of strain A4-2 after 50 generations (A4-2-50). The secondary mass spectrometry showed that the target protein sequence was detected in the bacterial precipitates. In the figure, "**" is used to indicate a significance level of *p* < 0.01.

The mRNA level of engineered *L. paracasei* was detected by RT-qPCR analysis, and the GLP-1 showed a higher expression in *L. paracasei* compared to the WT strain (Figure 4B). Furthermore, Nano LC-MS analysis confirmed the presence of the GLP-1 peptide in bacterial cell lysates, validating the correct translation of the integrated gene. The characteristic fragment EGQAAKEF was detected in the first generation of the engineered strain by Nano LC-MS analysis, absent in WT (Figure 4C).

### 3.5. Removal of Gene-Editing Plasmids

**Figure 5:**
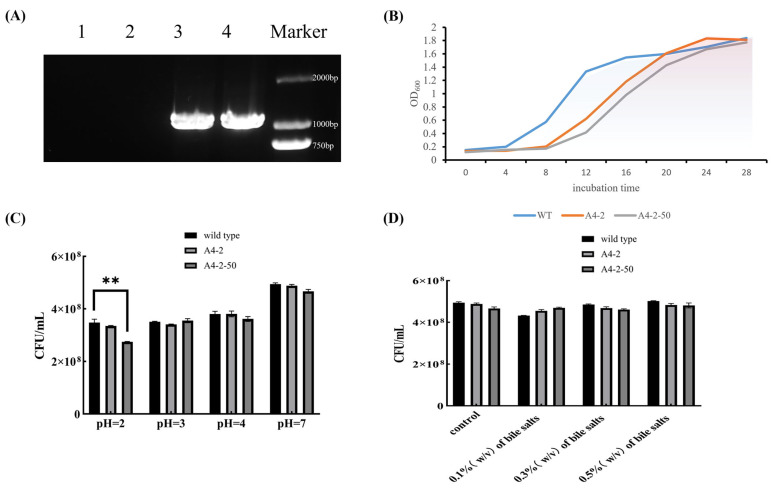
(**A**) The erythromycin gene was amplified by PCR with 1 (WT), 2 (pre-) and 3 (post-passaged genomes), and 4 (gene-editing plasmids) for electrophoresis detection. (**B**) The growth curves of the WT, gene-edited strain A4-2, and the gene-edited strain A4-2-50 after 50 generations of transmission. (**C**) The acid tolerance experiments of wild-type, gene-edited strain A4-2, and gene-edited strain A4-2-50 after 50 generations of transmission. (**D**) The bile salt tolerance experiment of wild-type, gene-edited strain A4-2, and gene-edited strain A4-2-50 (after 50 generations). In the figure, "**" is used to indicate a significance level of *p* < 0.01.

The A4-2 strain was subcultured 50 times in the MRS medium without erythromycin resistance. The A4-2-50 strain cannot grow on an erythromycin-resistant MRS medium, but it can grow normally on a normal MRS medium. The erythromycin gene was amplified by PCR with WT, post- and pre-passaged genomes and gene-editing plasmids, and detected using electrophoresis. The results show that there were obvious bands on the agarose electrophoresis pin pre-passaged genome and gene-edited plasmid, but the effective bands could not be observed in the post-passaged genome and WT (Figure 5A). These results suggest that the erythromycin gene in *L. paracasei* was successfully removed after subculture.

### 3.6. Basic Physiological Properties of Colonies

Compared with WT, the growth rate of gene-edited strain *L. paracasei* A4-2 was significantly lower after 6 h of cultivation. But no significant difference was observed in the OD_600_ of *L. paracasei* A4-2 compared with WT at the stable growth phase after 18 h cultivation (Figure 5B). The acid resistance and bile-salt resistance of *L. paracasei* A4-2 were also tested and no significant difference in growth was observed between the gene-edited strain and the original strain at the different pH values and bile salt concentrations exception for the pH 2 condition (Figure 5C,D). These results suggest that the gene-edited *L. paracasei* kept the resistance abilities to low pH conditions and bile-salt digestion.

### 3.7. Stable Expression of GLP-1 After Successive Passage

The stability of the gene-editing strain was also detected. The gene-editing strain A4-2 was continuously passed for 50 times. By sequencing, the fragments of P_ldh_ and *glp-1* were still firmly inserted into the *L. paracasei* genome, and no site mutation was detected in the two fragments in the successive strain A4-2-50 (Figure 4A). A4-2-50 also showed a high level of GLP-1 mRNA expression, but its expression was significantly downregulated compared to the first generation A4-2 (Figure 4B). Similarly, the production of GLP-1 peptide in A4-2-50 was validated by Nano LC-MS analysis. The peptide’s identity was verified through its mass and peptide fingerprint, and its sequence was still HAEGTFTSDVSSYLEGQAAKEFIAWLVKGR, aligning perfectly with theoretical aa sequences (Figure 4D). Furthermore, the growth of the successive strain was similar to that of the first-generation strain (Figure 5C), and the obvious resistance to acid and bile-salt were kept in A4-2-50 except at pH 2. The OD_600_ of A4-2-50 in pH 2 condition was significantly lower than the WT strain (Figure 5C,D). Taken together, the gene-editing strains could stably passage and express for 50 generations at least, and the key characteristics of development and metabolism also kept the stability. In this study, the expressed strains were developed by integrating *glp-1* gene in the genome of *L. paracasei* by gene editing. Subsequently, the GLP-1 peptide was successfully expressed in *L. paracasei*. In addition, the gene-editing strains could still stably express GLP-1 after subculturing for 50 times.

## 4. Discussion

As a promising drug delivery vector, *L. casei* has several advantages: Firstly, as a Gram-positive bacterium, it has natural biological safety because its cell wall does not contain endotoxin lipopolysaccharide. Secondly, it exhibits stronger gastrointestinal environment tolerance compared to similar strains, such as *Lactococcus lactococcus*. It can effectively adhere to intestinal epithelial cells and rapidly colonize in the intestinal tract. Thirdly, *L. paracasei* has unique biological characteristics in maintaining intestinal a microecological balance, regulating immune function, and promoting metabolic health. These properties make it an ideal chassis cell for building oral delivery systems to deliver active compounds such as GLP-1. It enables long-lasting delivery through intestinal colonization and synergistically exerts probiotic effects. It provides innovative solutions for the non-invasive treatment of diseases such as diabetes. This property confers a high degree of safety to *Lactobacilli*, making them highly sought after in the field of food science and medical research. Previously, Agarwal et al. successfully expressed GLP-1 using *L*. *lactis* [27].

At present, there are two primary strategies for applying of CRISPR-Cas9 in LAB, the exogenous SpyCas9 system and the endogenous CRISPR-Cas9 system. Fang et al. successfully achieved single- and double-gene knockout of *L. paracasei* CGMCC4691 using exogenous SpyCas9 [27], while Gu et al. used endogenous CRISPR-Cas9 of *L. paracasei* ATCC 27092 for gene editing [26]. However, exogenous SpyCas9 systems have disadvantages, such as partial inhibition of *Lactobacillus* growth and a reduction in transformation efficiency. The endogenous CRISPR-Cas9 systems, such as those based on the LpCas9 nuclease-encoded own gene in *L. casei*, not only improve the transformation efficiency by simplifying the complexity of traditional large vectors but also significantly enhance the stability of host receptors, providing a more reliable solution for long-term gene editing.

GLP-1 has shown great potential in the treatment of diabetes mellitus. However, the current injection method of drug delivery has caused significant inconvenience in patients′ daily lives. Frequent injections disrupt the daily routine of patients, and most patients could not maintain these in the long term due to the complexity of the administration process. As a result, this limits the broader adoption of GLP-1 therapy. So, it is urgent to develop an innovative delivery system for GLP-1 to simplify the therapy process and improve therapeutic efficacy. Agarwal et al. (2014) expressed GLP-1 in *L. lactococcus* by plasmid overexpression [27]. However, the expression plasmid is easy to lose due to the lack of selective pressure in the gastrointestinal tract when colonizing in the intestine [27]. In this study, the expression strains were developed by integrating *glp-1* gene in the genome of *L. paracasei* by gene editing. Subsequently, the GLP-1 peptide was successfully expressed in *L. paracasei*. In addition, the gene-editing strains could still stably express GLP-1 after subculturing for 50 times.

Gu et al. (2022) successfully transformed the exogenous gene into the genome of *L. paracasei* ATCC 27092 using the endogenous CRISPR-Cas9 system [26]. In the study, the chloramphenicol-resistant gene *cat* was integrated into the genome of *L. paracasei* by designing sgRNA and homologous repair templates, and then a positive clone was obtained on the chloramphenicol-resistant plate successfully. This result indicates that the gene was successfully expressed. In this study, the endogenous CRISPR-Cas9 system was also used to achieve the insertion of *glp-1* gene in *L. paracasei*, and the successful expression of GLP-1 was verified by RT-qPCR and Nano LC-MS. Both studies validated the reliability of the endogenous CRISPR-Cas9 editing system in *L. paracasei*, providing an important technical foundation for probiotic function research and industrial applications.

The efficiency of the CRISPR-Cas9 gene-editing system is highly dependent on the design of sgRNA and the physicochemical properties of the target sequence. The reason for the differences in the editing efficiency of two sgRNAs in this study may be because of the sequence characteristics of sgRNAs and secondary structure [28] and off-target effects [29]. Specifically, excessive GC content may lead to strong binding to DNA and reduce Cas9’s cleavage efficiency, while a low GC content may weaken binding stability [30]. The stem–loop structure of the sgRNA may fold and then bind to the Cas9 protein. This may hinder the formation of the normal Cas9-sgRNA complex. As a result, the editing efficiency was reduced [29]. The partial complementarity of sgRNAs with non-target sequences may lead to Cas9 binding to off-target points, reducing the concentration of Cas9 for effective editing. Repeating sequences near the target may competitively bind to the Cas9 complex, reducing the editing efficiency of the target point [30]. Simultaneously, different LAB strains have significantly different types of endogenous CRISPR systems (types I and II) and homologous recombination capabilities [31]. This limited the generality of the type II editing tool in this study. In addition, although the gene-edited strain stably expresses GLP-1 even after 50 passages, there are still gaps to be addressed in regard to long-term stability and safety. In the future, more attention can be devoted to the detection of long-term survival rates and tracking and monitoring the potential impact on the host microbiome [32].

In summary, this study represents a significant advancement in diabetes treatment by providing an engineered *L. paracasei* strain, which can integrate and stably express GLP-1. The *glp*-1 gene was successfully integrated into the genome of *L. paracasei* using an endogenous CRISPR-Cas9 system. Beyond diabetes, this technology holds promise for obesity management due to its appetite-suppressing properties. Moreover, *L. paracasei,* with sustained GLP-1 expression and functional activity, can also be applied in many fields such as oral microbiome interventions [33] and tumor immunotherapy [34].

## 5. Conclusions

In summary, this study utilized the endogenous CRISPR-Cas9 system to construct a gene-editing plasmid, achieving stable integration of the *glp-1* gene into the genome of *L*. *paracasei*. The expression of GLP-1 was verified through RT-qPCR and Nano LC-MS analysis, confirming the integration of the gene and the stable expression of the protein. It still shows good acid resistance, bile salt resistance and better genetic stability after continuous passaging. Combined with previous studies, these results confirm the reliability of the endogenous CRISPR-Cas9 editing system in *L*. *paracasei* and establish a key technical foundation for probiotic engineering and transformative applications.

## Figures and Tables

**Table 1:** Bacterial strains and plasmids used in this study.

Title	Features	Source
Trelief ^®^ 5α Chemically	*E. coli* clone	Tsing ke Biotechnology Co., Ltd. Beijing, China
* L.paracaise NMG-13 *	*Lactobacillus paracasei*, strain to be edited	Laboratory storage
pTRST	pTRKH2-Amp derived plasmid, backbone plasmid incorporating P_ldh_-sgRNA and LpCas9 Scaffold element	Laboratory storage
pTRST-sgRNA-A	Derived from pTRST with the addition of sgRNA	This experiment
pTRST-sgRNA-B	Derived from pTRST with the addition of sgRNA	This experiment
pTRST-sgRNA-A1	Derived from pTRST-sgRNA-A with the addition of the P_ldh_ promoter	This experiment
pTRST-sgRNA-B1	Derived from pTRST-sgRNA-B with the addition of the P_ldh_ promoter	This experiment
pTRST- GLP-A	Derived from pTRST-sgRNA-A1 with the addition of glp-1 and homology arms	This experiment
pTRST- GLP-B	Derived from pTRST-sgRNA-B1 with the addition of glp-1 and homology arms	This experiment

**Table 2:** Primers used in this study.

Primers	Sequences
SgRNA-A	CTAGAGTTGTTTCACATCGTTCCGGC
SgRNA-B	CTAGACCGCAAGTCCTTCTACAATGC
pTRST-A-F	CTAGAGTTGTTTCACATCGTTCCGGC
pTRST-A-R	AGACGCCGGAACGATGTGAAACAACT
KZ-Pldh -F	ATGTGAAAGCAATCGACTAACCATACCCATGGACGGATTTT
KZ-Pldh-R	AATGTGCCTTCAGCATGCATAGGTGATATCATCCTTTCTTATGTGC
PD-GLP-R	TTAACGGCCCTTGACCAACC
YZ-DPD-F1	CAGGTAGCGAACTACACGT
HR1-F	GTTGGGCCATACATTTTTTTCAAGGGCAAGTTTGGCGC
HR1-R	CTTCAGCATGCATTTAGTCGATTGCTTTCACATTGTAGA
DPD-F1	GCCCATGTTGGGCCATACATTTTTTTCAAGGGCAAGTTTGGCGC
DPD-R1	CAGTGAATTCCCGGGGATCCATATACCACAGGCCACGATTGC
HR2-F	GTCAAGGGCCGTTAATTTGCTTAATTAGTTGGCAGCTTG
HR2-R	CAGTGAATTCCCGGGGATCCATATACCACAGGCCACGATTGC
pTRST-B-F	CTAGACCGCAAGTCCTTCTACAATGC
ZTKZ-F	GGATCCCCGGGAATTCACT
ZTKZ-R	AAAAAAATGTATGGCCCAACATG
YZ-QR-F	TACAAGCCAGGCGACGACAT
YZ-QR-R	TAGCAGCTTGGCCCTCCAAGT
pTRST-B-R	AGACGCATTGTAGAAGGACTTGCGGT
RNA-A-F	ATGCATGCTGAAGGCACATTT
RNA-A-R	TTAACGGCCCTTGACCAACCAA
PD-16S-F	CCTCCAACACCTAGCATTCAT
PD-16S-R	TGTAACTGACGCTGAGGCT

**Table 3:** The candidate SgRNA of LpCas9-targeting *eno*.

No.	PAM	Cut Position	Strand	Guide Sequence	On-Target Score	Off-Target Score
A	tgaaa	1113	+	gttgtttcacatcgttccgg	42.6	100.0
B	tgaaa	1286	+	ccgcaagtccttctacaatg	40.3	100.0
C	tgaaa	1108	−	tcagtttcaccggaacgatg	35.6	99.5
D	tgaaa	1532	+	tttagcgaggtcgcttgggt	7.1	100.0
E	tgaaa	1615	+	atactagggataagcacaaa	16.3	100.0

+ represents the Coding Strand, − represents the Non-coding Strand.

## Data Availability

The original contributions presented in the study are included in the article, further inquiries can be directed to the corresponding authors.
